# *Lactobacillus* Probiotics Improve Vaginal Dysbiosis in Asymptomatic Women

**DOI:** 10.3390/nu15081862

**Published:** 2023-04-13

**Authors:** AbuZar Ansari, Dooheon Son, Young Min Hur, Sunwha Park, Young-Ah You, Soo Min Kim, Gain Lee, Seungbeom Kang, Yusook Chung, Sanghyun Lim, Young Ju Kim

**Affiliations:** 1 Department of Obstetrics and Gynecology and Ewha Medical Research Institute, College of Medicine, Ewha Womans University, Seoul 07984, Republic of Korea; 2 Graduate Program in System Health Science and Engineering, Ewha Womans University, Seoul 07984, Republic of Korea; 3 R&D Center, Cell Biotech Co., Ltd., Gimpo 10003, Republic of Korea

**Keywords:** *Lactobacillus*, probiotics, cervicovaginal fluid, vaginal microbiota, vaginal dysbiosis, bacterial vaginosis, Nugent score

## Abstract

Vaginal dysbiosis can lead to serious infections in asymptomatic women. *Lactobacillus* probiotics (LBPs) are being investigated as a promising therapy for reversing vaginal microbiota dysbiosis. This study aimed to investigate whether administering LBPs could improve vaginal dysbiosis and facilitate the colonization of *Lactobacillus* species in asymptomatic women. 36 asymptomatic women were classified based on the Nugent score as Low-NS (n = 26) and High-NS (n = 10) groups. A combination of *Lactobacillus acidophilus* CBT LA1, *Lactobacillus rhamnosus* CBT LR5, and *Lactobacillus reuteri* CBT LU4 was administered orally for 6 weeks. The study found that among women with a High-NS, 60% showed improved vaginal dysbiosis with a Low-NS after LBP intake, while four retained a High-NS. Among women with a Low-NS, 11.5 % switched to a High-NS. Genera associated with vaginal dysbiosis were positively correlated with the alpha diversity or NS, while a negative correlation was observed between *Lactobacillus* and the alpha diversity and with the NS. Vaginal dysbiosis in asymptomatic women with an HNS improved after 6 weeks of LBP intake, and qRT-PCR revealed the colonization of *Lactobacillus* spp. in the vagina. These results suggested that oral administration of this LBP could improve vaginal health in asymptomatic women with an HNS.

## 1. Introduction

The composition of the vaginal microbiota is associated with beneficial or detrimental effects on vaginal health [1,2]. Vaginal microbiota ecology, especially the high relative abundance of *Lactobacillus* spp., is considered crucial for vaginal health in preventing the invasion of pathogens and decreasing susceptibility to gynecological infections, such as sexually transmitted infections (STIs), including bacterial vaginosis (BV) [3,4,5]. *Lactobacillus* spp. produces lactic acid via metabolic processes, thus, contributes to an acidic environment (low pH) in the vagina, where they exert antimicrobial, antiviral, and immunomodulatory effects [6]. *Lactobacillus crispatus*, *Lactobacillus gasseri*, *Lactobacillus iners*, and *Lactobacillus jensenii* are the most frequently detected microbial species in a healthy vagina and may be involved in the prevention of pathogenic infections by maintaining richness in the vaginal microbiota’s abundance [7]. A high abundance of *Lactobacillus* spp. in the vaginal microbiota indicates vaginal eubiosis and is associated with a healthy vaginal environment [8,9]. In contrast, vaginal dysbiosis is characterized by decreased levels of *Lactobacillus* spp. and increased levels of anaerobic bacteria, primarily *Gardnerella* spp., *Atopobium* spp., *Prevotella* spp., and *Ureaplasma* spp. [10,11,12]. Vaginal dysbiosis dynamics can lead to a significant increase in the risk of BV, preterm birth, and urinary tract infections [13,14,15,16]. In terms of BV, the following eight genera have been reported to be associated with vaginal dysbiosis: *Gardnerella*, *Atopobium*, *Megasphaera*, *Eggerthella*, *Aerococcus*, *Leptotrichia/Sneathia*, *Prevotella*, and *Papillibacter* [17].

Probiotics are “living microorganisms” that have beneficial health effects when consumed in appropriate amounts, as defined by the World Health Organization and the United Nations Food and Agriculture Organization in 2002 [18,19]. Probiotic intake plays a role in maintaining and improving the diversity and dynamics of the gut microbiota profile [20,21]. In addition, probiotics prevent diseases through functions such as stabilizing the gut microbiota, generating short-chain fatty acids, and inhibiting the settlement of pathogenic microbiota [22]. The gut microbiota significantly influences metabolic pathways of distant organs, including reproductive homeostasis [23,24,25]. Although the gut microbiota and vaginal microbiota represent two different ecosystems, the gut microbiota is considered an extravaginal reservoir that might influence the risk of vaginal dysbiosis through dysbiotic gut microbiota [26]. Specific probiotics, such as *Lactobacillus* probiotics (LBPs), are suggested to have safe and promising therapeutic effects to modulate microbiota homeostasis in the general population [27]. Studies have shown that oral LBP intake not only improves vaginal microbiota dysbiosis significantly, but also improves leucorrhea, itching, and vulvo-vaginal erythema/edema [28,29]. In addition, *Lactobacillus* surface active molecules (peptidoglycan, lipoteichoic acid, exopolysaccharides, etc.) antagonize the pathogenic microbes [30]. Therefore, specific LBP combinations could be novel treatments against pathogenic microbes that cause vaginal dysbiosis by increasing *Lactobacillus* abundance.

Symptomatic vaginal dysbiosis, such as the STI BV, is characterized by symptoms such as vulvovaginal itching, burning, irritation, bad odor, rashes, unusual discharge, and pain in the vagina [5]. In addition, approximately half of BV-positive women have no clear symptoms and are considered to have asymptomatic vaginal dysbiosis [31,32]. The Nugent score (NS) is used as the gold standard tool for screening for asymptomatic BV and identifying suitable treatments, such as antibiotics [33,34]. Limited data exist concerning the effect of LBPs and effectiveness of molecular-based diagnosis methods, such as STI-PCR and 16S rRNA amplicon sequencing, in asymptomatic vaginal dysbiosis [35,36,37,38]. To obtain accurate information on pathogenicity, molecular-based diagnostic validation can better confirm a diagnosis and improve understanding of how to manage a microbial dysbiosis. In the present study, it was hypothesized that LBP intake could improve vaginal dysbiosis and facilitate the colonization of *Lactobacillus* spp. in the vagina in women with asymptomatic vaginal dysbiosis. To investigate whether the LBP modulates vaginal dysbiosis and changes the microbiota dynamics to improve vaginal dysbiosis and maintain a normal vaginal environment, the NS was used to first categorize the study subjects. Then, 16S rRNA gene amplicon microbiota analysis (NGS) and quantitative real-time polymerase chain reaction (qRT-PCR) were performed for validation of the vaginal microbiota in cervicovaginal fluid (CVF) samples of women with asymptomatic vaginal dysbiosis.

## 2. Materials and Methods

### 2.1. Enrolled Subject Criteria and Sample Collection

Overall, 57 premenopausal women aged between 19 and 55 years old who visited the Obstetrics and Gynecology outpatient clinic or the Health Examination Center at Ewha Womans University Mokdong Hospital from June 2021 to September 2021 were enrolled. Several subjects were excluded based on the following exclusion criteria: undergoing immune or hormonal therapy; taking probiotics or antibiotics; having a history of alcohol or drug addiction; or being at risk of pregnancy. Finally, after counseling, women who agreed to participate in the trial/study provided written informed consent prior to enrolment.

The CVF samples of the participants were collected using an NBG-S5V Swab kit (Noble Biosciences, Suwon-si, Gyeonggi-Do, Republic of Korea) by swabbing the exocervix, and the swabs were then dipped into buffer. Collected samples were immediately transferred to a laboratory and stored at −80 °C for further microbiome analysis. The CVF samples were stored under strict regulations for research and analysis institutes.

The Institutional Research Board (IRB) of Ewha Womans University (Mokdong Hospital, Seoul, Republic of Korea) approved this prospective study (IRB approval no. 2020-11-035-007) of LBP oral intake by healthy women with asymptomatic vaginal dysbiosis. The study was conducted in accordance with the approved guidelines.

### 2.2. Probiotic Combination and Intervention

The LBP used in the study contained a combination of *Lactobacillus acidophilus* CBT LA1 (LA1, KCTC 11906BP), *Lactobacillus rhamnosus* CBT LR5 (LR5, KCTC 12202BP), and *Lactobacillus reuteri* CTB LU4 (LU4, KCTC 12397BP) strains isolated from Korean human feces by Cell Biotech (Gimpo-si, Republic of Korea). The CBT of LA1, LR5, and LU4 was prepared with dextrose, fructooligosaccharide, xylitol, pomegranate powder, pomegranate spice, malic acid, corn starch, and Starch 1500, which are known as safe materials used in the preparation of various asymptomatic functional foods.

The intake method was one packet of probiotics (2 g) per day, which contained 1 × 10^10^ CFU of total bacterial strains, either directly or with water. After the collection of CVF on the first visit (the initial visit), probiotics were administered for 3 weeks, and Gram staining, NGS, and qRT-PCR were performed. After 3 weeks, i.e., on the second visit (mid-visit), CVF samples were again collected, probiotics were administered for another 3 weeks, and Gram staining, NGS, and qRT-PCR were performed on the collected sample. Finally, on the third visit (the final visit), which was the endpoint of the study, CVF samples were collected, and Gram staining, NGS, qRT-PCR, and STI-PCR (only on the third visit) were performed on the collected sample. In addition, on the second and third visits, the subject’s medication compilation was recorded after LBP intake.

**Figure 1:**
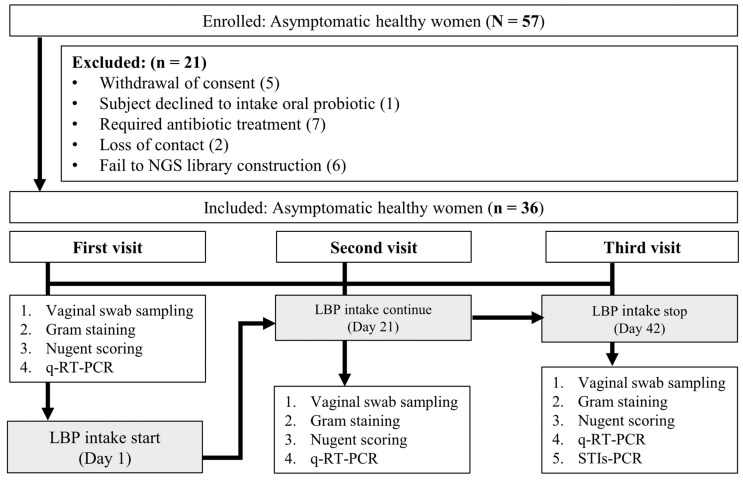
Flow chart of subject analysis. qRT-PCR, quantitative real-time polymerase chain reaction; LBP, *Lactobacillus* probiotic; STI-PCR, sexually transmitted infection polymerase chain reaction.

The study subjects who were diagnosed with vaginitis according to the Gram stain results of the CVF sample collected on the first visit and ones who needed treatment were subjected to additional testing with the genital mycoplasma culture or culture and sensitivity test. The subjects were then guided to appropriate treatment and were not included in the study. In total, 142 samples were collected from 57 women across three visits; however, only 108 samples from 36 women were included in the final analysis (Figure 1).

### 2.3. Gram Staining and NS

For Gram staining, one drop of CVF sample was smeared on a glass slide, dried, stained with crystal violet for 1–2 min, and bleached, after which the slides were observed under a microscope for the presence of gram-positive bacteria. The presence and type of microorganisms in the vagina were examined to compare the microbiota present before and after LBP intake. Therefore, not only were changes in the microorganisms in the vagina noted, but also whether the vaginal environment was asymptomatic or more vulnerable to vaginitis or other infections/diseases. Slide smears were examined by three independent microbiologists who scored and interpreted the slides independently using the NS method. Using the Gram stain of CVF samples, the NS was applied as follows: 0–3, normal; 4–6, intermediate; and 7–10, BV. Based on the NS at first visit, the study subjects with a Low NS (LNS ≤ 3) were designated as Group A (normal group), and those with a High NS (HNS ≥ 4–10) were designated as Group B (abnormal group).

### 2.4. DNA Extraction and STI-PCR

For STI-PCR, the CVF samples were vigorously agitated in a buffer to dislodge the cells. Microbial DNA was extracted using the FastDNA SPIN Kit for Soil (MP Biochemicals, Santa Ana, CA, USA) according to the manufacturer’s instructions. The extracted microbial DNA was purified using a DNeasy PowerClean Pro Cleanup Kit (Qiagen, Hilden, Germany), and the DNA quality was assessed using a QuickDrop (Molecular Devices, San Jose, CA, USA). The concentration of the purified DNA was measured using the Qubit dsDNA BR Assay kit (Thermo Fisher Scientific, Waltham, MA, USA).

### 2.5. V4–V5 Targeted 16S rRNA Gene Sequencing

Sequencing of 16S rRNA was performed to determine the relative abundance of *Lactobacillus* spp. and to assess the shift in the presence and type of each bacterial species before and after LBP intake. Extracted DNA was used for PCR amplification. The amplicon (5 µL) of each participant was subjected to electrophoretic separation on a 1.5% agarose gel (Cosmogentech, Ltd., Seoul, Republic of Korea), and the product (~490 bp) was visualized under UV light (Daihan Scientific, Ltd., Wonju, Republic of Korea). A sequencing library with subsequent steps (purification, sample indexing, sample quantification, and pooling) was prepared according to the Illumina 16S metagenomic sequencing library preparation guide (Illumina, San Diego, CA, USA). The V4–V5 region of the bacterial 16S rRNA gene was amplified using 16S rRNA gene sequencing using the following primers: a forward primer in the V4 region (CCA GCM GCC GCG GTA ATW C) and a reverse primer in the V5 region (CC GTC AAT TYY TTT RAG TTT). The amplified sequencing library was purified using Agencourt^®^ AMPure XP beads (Beckman Coulter, Brea, CA, USA), and the quality of the library was assessed using a 2100 Bioanalyzer (Agilent, Santa Clara, CA, USA). The library pool was sequenced with 250 bp paired-end reads on the MiSeq platform (Illumina, San Diego, CA, USA) using the MiSeq reagent kit V2 (Illumina, San Diego, CA, USA).

### 2.6. qRT-PCR

qRT-PCR was performed to confirm whether the three *Lactobacillus* spp. in the LBP colonized the vagina or not. After culturing the three bacterial strains that were ingested by the subject, the cells were counted and used as a standard for the experiment. Then, DNA was extracted using the FastDNA SPIN Kit for Soil (MP Bio-chemicals) following the manufacturer’s instructions. The extracted microbial DNA was purified using the DNeasy PowerClean Pro Cleanup Kit (Qiagen), and the DNA quality was assessed using QuickDrop (Molecular Devices). The concentration of the purified DNA was measured using the Qubit dsDNA BR Assay kit (Thermo Fisher Scientific). Purified DNA was used as a qRT-PCR standard. Values of cells/mL were converted to DNA concentration values (cells/ng) and used for calculations. Prime Q-Master mix (Genet Bio, Chungnam, Republic of Korea) was used for qRT-PCR. The primers used for qRT-PCR are listed in Supplementary Table S1 and the amplification conditions are listed in Supplementary Table S2.

### 2.7. Data Processing and Statistical Analysis

Targeted sequencing of the V4–V5 region of the microbiota was performed using Illumina MiSeq. Raw sequencing data were processed using the Quantitative Insight Into Microbial Ecology software package 2 (QIIME2, v2021.11, http://qiime2.org, accessed on 6 February 2023). Denoising was performed using DADA2 and a taxonomy table was created using the SILVA database (v138). Data were normalized to a depth of 14,000, which was the minimum depth of the sample that was used for alpha (amplicon sequence variants [ASVs], Shannon diversity, and Pielou’s evenness) and beta diversity analyses. The results following data processing, analysis, and visualization were analyzed using the ggplot2 package of R (v4.1.3), and statistical analysis was performed using the Wilcoxon rank-sum test, Wilcoxon signed-rank test, Kruskal–Wallis test, Mann–Whitney test, and PERMANOVA using the vegan package. Linear discriminant analysis effect size was performed using Galaxy (https://huttenhower.sph.harvard.edu/galaxy. accessed on 6 February 2023). The data were then filtered and normalized. For clinical parameter statistical analysis, the two-tailed p-value was applied, and a *p*-value < 0.05 was considered to be significant.

## 3. Results

### 3.1. Demographic Profile, Gram Stain, and NS

A total of 57 asymptomatic women aged 19–55 years were enrolled in this study. Of the 57 subjects, five withdrew their consent, one declined to take the oral LBP, seven had to be treated with antibiotics, two were lost to follow-up, and six had samples that failed during NGS library construction. Therefore, only 36 women were included in the final analysis. The study subjects with an LNS ≤ 3 were designated as the normal group, and those with an HNS ≥ 4–10 were designated as the abnormal group, after analysis of the NS of the Gram stain results performed at the first visit (Table 1). The median age was 41.0 years, and the median BMI was 23.2 on the first visit for all 36 subjects. There were no significant differences in age or BMI between the two groups. In the LNS group (n = 26), four women had positive gram stains on the second visit, and three women had positive gram stains on the third visit, whereas in the HNS group (n = 10), four women had positive gram stains on the second and third visits (Table 1). There was a 60% reduction in the HNS group (n = 6) after LBP intake. Concerning the NS, at the first visit, the difference was significantly high (*p* < 0.001) between groups, while the difference decreased at the second visit (*p* < 0.04) and the third visit (*p* < 0.06) with the decrement of the NS after LBP intake.

### 3.2. Alpha Diversity and Vaginal Microbiota Taxonomy of the Normal and Abnormal Groups

**Figure 2:**
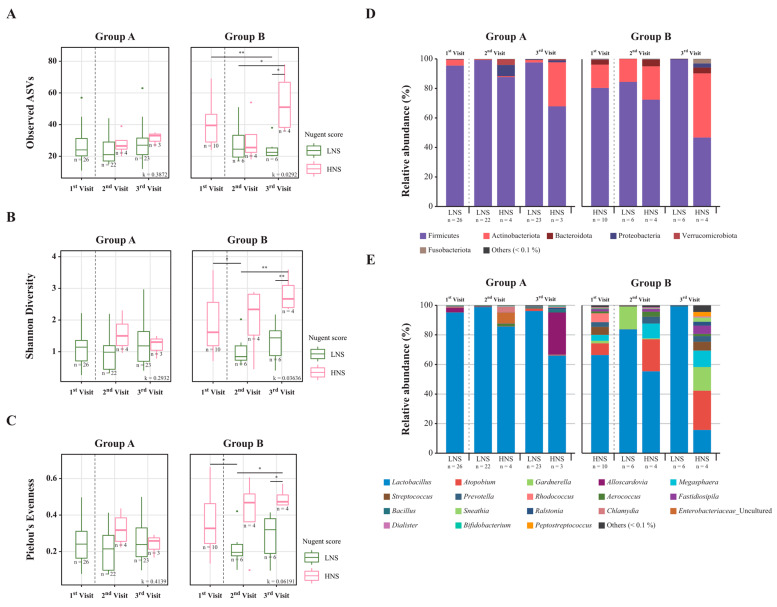
Changes in alpha diversity and taxonomy composition for each visit in the two groups divided by the Nugent score of the first visit. Boxplot showing (**A**) observed amplicon sequence variants (ASVs), (**B**) Shannon diversity, and (**C**) Pielou’s evenness in Group A and B. Relative abundance at the (**D**) phylum and (**E**) genus levels in Group A and B. Each group was classified as LNS or HNS based on the NS at the first visit. * *p* < 0.05, ** *p* < 0.01 (Wilcoxon rank-sum test). An average of <1% was labeled as other.

Amplicon sequencing of 16S rRNA was performed to compare the taxonomic composition of the vaginal microbiota in the samples collected on the first, second, and third visits. Alpha diversity was measured using three indices: observed ASVs, Shannon diversity, and Pielou’s evenness, at the different visits. At the initial visit, samples classified as LNS were categorized into Group A, and samples classified as HNS were categorized into Group B. It was confirmed that in Group B, samples with a decreased NS (samples that changed from an HNS to an LNS) showed a significant difference in alpha diversity as the number of visits increased. Furthermore, it was observed that the alpha diversity values of 60% of the samples that changed from an HNS in Group B to an LNS became similar to those of Group A; however, for the LNS, there was no significant difference between the visits at any indices of alpha diversity (Figure 2A–C). Observably, the indices of alpha diversity decreased significantly in six women with an HNS who were classified as having an LNS on the second and third visits (Figure 2A–C).

The taxonomic composition of six phyla in the vaginal microbiota: Firmicutes, Actinobacteria, Bacteroidota, Proteobacteria, Verrucomicrobiota, and Fusobacteriota, was observed (>1%) at each visit. It was found that Firmicutes were the dominant bacteria in most of the vaginal samples analyzed. Additionally, it was observed that Actinobacteriota were predominantly dominant in HNS samples (Figure 2D). At the genus level, 17 genera: *Lactobacillus, Atopobium, Megasphaera*, *Prevotella*, *Alloscardovia*, *Rhodococcus*, *Gardnerella*, *Streptococcus*, *Bacillus*, *Aerococcus*, *Fastidiosipila*, *Enterobacteriaceae*, *Ralstonia*, *Chlamydia*, *Sneathia*, *Dialister,* and *Peptostreptococcus*, were observed (>1%) on all visits (Figure 2E). The relative abundance of the genus *Lactobacillus* belonging to the phylum Firmicutes was assessed in all samples from Groups A and B. After LBP oral intake, in Group B, six samples that changed from HNS to LNS were dominated by *Lactobacillus* at the second and third visits, while three samples in Group A that changed from LNS to HNS at the third visit showed that the relative abundance of *Lactobacillus* decreased and the ecosystem appeared destroyed, but alpha diversity did not increase significantly (Figure 2E).

### 3.3. Correlation between NS and the Vaginal Microbiota Taxonomy of Normal, Intermediate, and BV Groups

**Figure 3:**
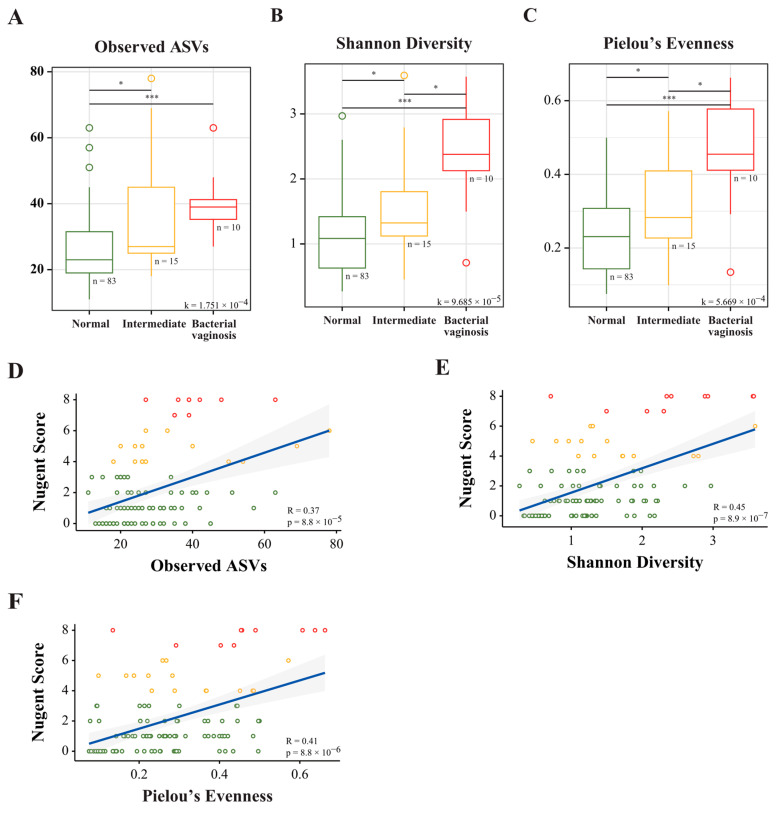

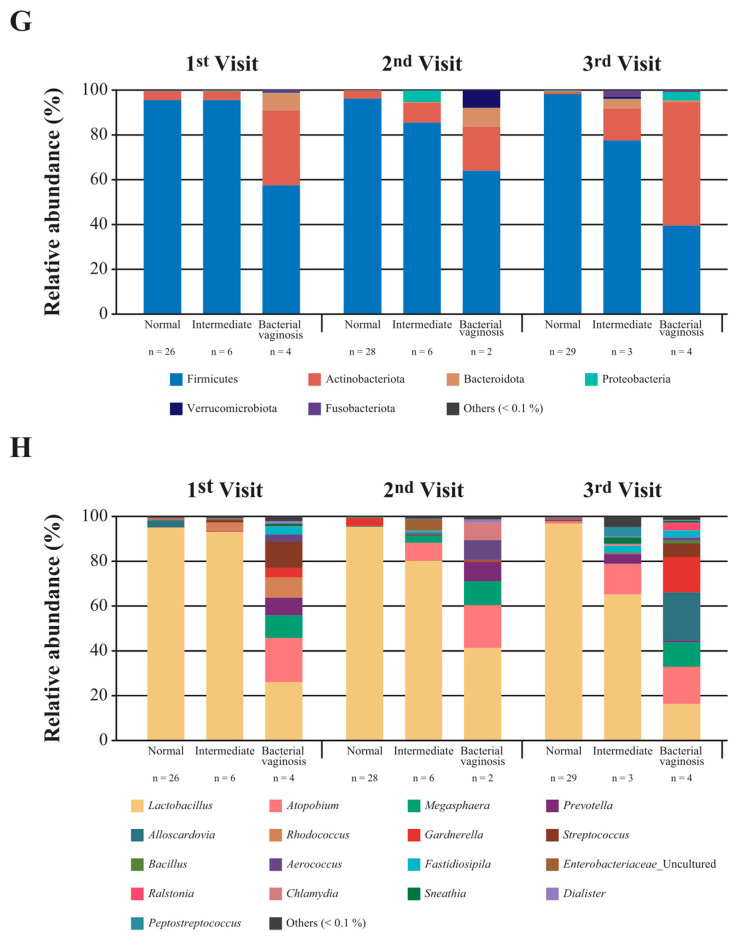
Alpha diversity and correlation between the Nugent score and alpha diversity, and the taxonomic composition of the vaginal microbiota between groups at different visits. Boxplot illustrating the (**A**) observed amplicon sequence variants (ASVs), (**B**) Shannon diversity, and (**C**) Pielou’s evenness. Spearman’s correlation between the Nugent score with the (**D**) observed ASVs, (**E**) Shannon diversity, and (**F**) Pielou’s evenness. Relative abundance by visit order at the (**G**) phylum and (**H**) genus levels. * *p* < 0.05, *** *p* < 0.001 (Wilcoxon rank-sum test).

The correlation between alpha diversity and the NS was analyzed to compare the composition of microbiota as assessed by the NS and 16S rRNA amplicon sequencing. The HNS group was further classified into intermediate and BV groups, and the LNS group was considered the normal group. The mean alpha diversity indices of the intermediate and BV groups were significantly different from those of the LNS group (*p* < 0.05; Figure 3A–C). Moreover, there was a positive correlation between the NS and alpha diversity for each index of alpha diversity (*p* < 0.05; Figure 3D–F). At the phylum level, a high abundance of Firmicutes (~>80–90%) was observed in the normal and intermediate groups compared to that in the BV group (>40–60%) (Figure 3G). At the genus level, *Lactobacillus* was dominant at >90% and slightly improved among visits in the normal group after LBP intake. The abundance of *Lactobacillus* in the intermediate group was ~70–90%, while that in the BV group was ~20–40% due to the increased abundance of other genera across all visits compared with that in the normal group (Figure 3H). The genera *Atopobium*, *Megasphaera*, *Prevotella*, *Gardnerella,* and *Streptococcus* were highly distributed in the BV group (Figure 3H).

### 3.4. Correlation between Alpha Diversity and the NS with Dominant Vaginal Microbiota

**Figure 4:**
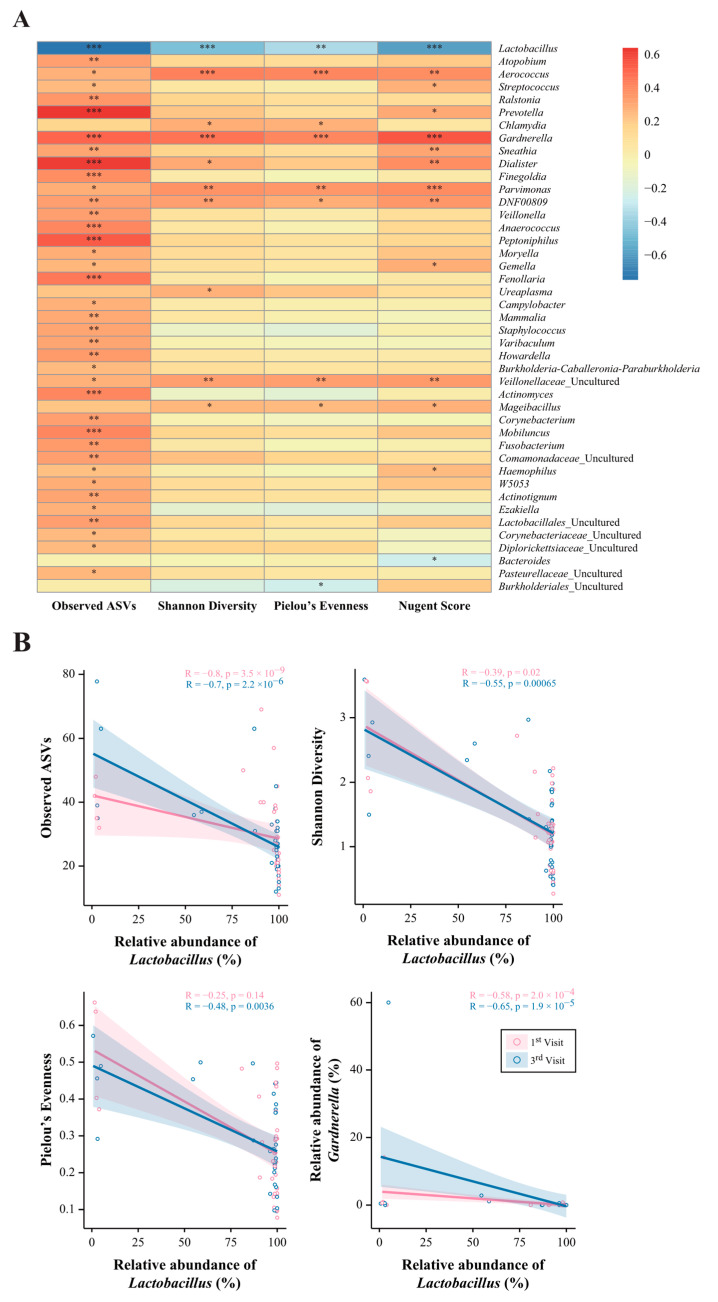
Correlation between parameters, including alpha diversity, Nugent score, and vaginal microbiota at the first and third visits. (**A**) Heatmap showing the correlation between parameters and the vaginal microbiota. (**B**) The correlation between parameters and the abundance of *Lactobacillus* at the first and third visits. * *p* < 0.05, ** *p* < 0.01, *** *p* < 0.001.

A correlation analysis was performed between the parameters alpha-diversity, NS, and vaginal microbiota, and a heatmap was constructed. A positive correlation was observed between all indices of alpha diversity and dominant vaginal microbiota, such as *Aerococcus*, *Gardnerella*, *Parvimonas*, DNF00809, *Veillonellaceae*, and *Mageibacillus*. In addition, genera *Prevotella*, *Sneathia*, *Dialister*, *Anaerococcus*, *Peptoniphilus*, *Fenollaria*, *Actinomyces*, and *Mobiluncus* showed a highly significant positive correlation for the ASV index of alpha diversity only (*p* < 0.01). As well as *Aerococcus*, *Prevotella*, *Gardnerella*, *Sneathia*, *Dialister*, *Parvimonas*, *DNF00809*, *Veillonellaceae*, and *Mageibacillus*, three genera, *Streptococcus, Gemella,* and *Haemophilus*, showed a significant positive correlation with the NS (*p* < 0.05). As expected, *Lactobacillus* showed a significant negative correlation with all indices of alpha diversity and the NS (*p* < 0.05) (Figure 4A). Concerning a highly significant negative correlation of beneficial *Lactobacillus* and a highly significant positive correlation of pathogenic *Gardnerella* with alpha diversity and NS, correlation analysis was performed with the first and third visits (*p* < 0.001) (Figure 4B). The correlation between *Lactobacillus* and parameters, including Shannon diversity, Pielou’s evenness, and *Gardnerella*, at the third visit showed much greater significance than that at the first visit. It was evident that LBP intake resulted in a significant effect on asymptomatic vaginal dysbiosis (Figure 4B).

### 3.5. Beta Diversity and Microbiota Shift after 6 Weeks of LBP Intake

**Figure 5:**
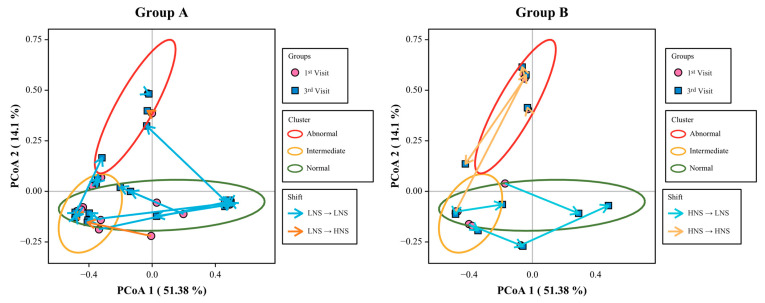
Beta diversity and shift after 6 weeks of LBP intake. The principal coordinate analysis illustrates the beta diversity shift in Group A and B. Arrows represent the shift from the first visit to the third visit. Group A, LNS at first visit; Group B, HNS at first visit. PCoA, principal coordinate analysis.

Changes in the vaginal microbiota composition between the normal, intermediate, and BV groups were analyzed after six weeks of LBP intake using Bray–Curtis dissimilarity. Clusters were observed to demonstrate dissimilarity between the normal, intermediate, and BV groups (Supplementary Figure S1). In the group-wise analysis of the first and third visits, a pattern was observed after LBP intake; a shift from LNS to HNS clusters was observed in two of the 26 women in the LNS group (normal) (Figure 5A). Whereas in the HNS (abnormal), six of the 10 women showed a shift from HNS to LNS clusters (Figure 5B). The arrow represents the shift from the first to the third visit.

### 3.6. Quantitative Expression of Lactobacillus Species

**Figure 6:**
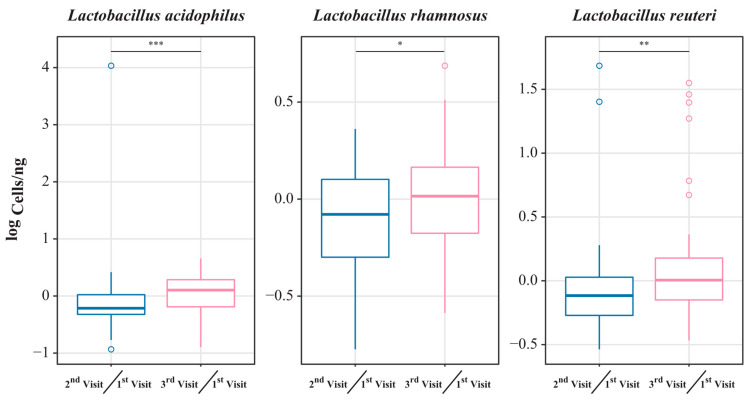
Expression of *Lactobacillus* spp. present in the oral probiotic in cervicovaginal fluid samples, as assessed by qRT-PCR. The expression ratio at the second and third visit compared to that of the first visit. qRT-PCR quantification was expressed as log cells/ng. * *p* < 0.05, ** *p* < 0.01, *** *p* < 0.001 (Wilcoxon signed-rank test).

qRT-PCR was performed to assess differences in the transcripts of probiotic *Lactobacillus* spp. in CVF samples among the three visits. The ratio of expression was calculated by dividing the expression in CVF samples collected on the second and third visits by that collected on the first visit. After LBP intake, by comparing the expression ratio of the ingested species, it was found that the total cell of *L. acidophilus* (*p* < 0.001), *L. rhamnosus* (*p* < 0.05), and *L. reuteri* (*p* = 0.01) increased significantly (Figure 6). Overall, the results showed that probiotic *Lactobacillus* spp. colonized the vagina after LBP intake. In Group B, the majority of samples showed a downward shift on PCoA2 (y-axis) and a rightward shift on PCoA1 (x-axis).

## 4. Discussion

Vaginal microbiota diversity is important for maintaining vaginal health and preventing disease, and this fluctuates with ethnicity, age, and lifestyle [39]. In addition, vaginal microbiota diversity fluctuates with hormone levels from puberty to menopause [40]. According to the World Health Organization, in women of reproductive age (15–49 years), *Lactobacillus* spp. is the dominant species in the vagina [12]. Herein, the study subjects were asymptomatic women with vaginal dysbiosis aged between 19 and 55 years. Their vaginal health was analyzed, and the improvement of vaginal dysbiosis by LBP intake for 6 weeks was evaluated (Figure 1). After applying the exclusion criteria, 36 asymptomatic women were divided into two groups: the LNS group (n = 26; as normal) and the HNS group (n = 10; as abnormal), after determining the NS on the first visit. The study aimed to investigate the status of vaginal dysbiosis of asymptomatic women using the NS and validate it using molecular diagnostic tools through 16S rRNA sequencing and qRT-PCR in CVF samples. The results suggested a potential therapeutic effect of LBP intake on vaginal health improvement over the 6-week intervention period through decreased NS and increased colonization of *Lactobacillus* spp. in the vagina.

As discussed previously, the NS is considered the gold standard technique for evaluating BV status, especially during pregnancy [34,41]. In this study, the NS was used to group the asymptomatic women into the LNS (n = 26) and HNS (n = 10) groups, and vaginal dysbiosis was evaluated after 6 weeks of LBP intake. More than 27% (n = 10) of women fell into the HNS group showing asymptomatic vaginal dysbiosis at the first visit, and 60% (n = 6) of that 27% shifted to the LNS group after 6 weeks of LBP intake, as revealed through Gram staining (Figure 2). The sample size of 10 individuals in Group B may not have been representative of the entire population of the HNS group. But Group B was a divided group among 36 asymptomatic women, and 10 out of 36, or 28%, were applicable. The effect needs to be verified by making changes in future studies, such as using a larger number of samples, including patients with vaginitis, and having a longer period. However, the LNS group had a high relative abundance of *Lactobacillus* spp. before LBP intake, which was slightly changed after intake as three women in the LNS group shifted to the HNS group. This was potentially due to host hormonal or immunological factors or metabolic pathway differences [42,43,44]. In a shift from an HNS to an LNS, the vaginal microbiota diversity decreases with an increased relative abundance of *Lactobacillus*, which is an indication of vaginal dysbiosis normalization [45]. As observed, the abundance of *Lactobacillus* spp. was high (>90%) in the LNS group, and the high abundance of *Gardnerella* spp. in the HNS group suggested potential for the diagnosis of BV and a correlation with the NS [17,46]. The improvement in the abundance of *Lactobacillus* spp. in the vaginal microbiota from an HNS to an LNS, i.e., a normal condition, might have been due to the translocation of *Lactobacillus* from the gut to the vagina after intake of the LBP.

The normal vaginal microbiota is composed of gram-positive bacilli (Doderlein’s bacilli), which are thought to translocate from the gut, and are of the genus *Lactobacillus* [45,47]. The six common bacterial phyla were Firmicutes, Bacteroidetes, Proteobacteria, Actinobacteria, and Fusobacteria, with a high relative abundance of *Lactobacillus* spp. present in both the gut and vagina, as observed through vaginal microbiota analysis [40,48,49,50]. The *Lactobacillus* spp. can colonize the vagina after oral administration through the gut pathway [51]. In a previous study, a combination of *L. rhamnosus* GR-1 and *L. reuteri* RC-14 was administered as an LBP, and four different samples (buccal mucosa, tongue coat, feces, and vagina) were analyzed. The longitudinal dataset revealed that there was very limited probiotic translocation to the vagina [52]. Although LBP spp. were detected more frequently in the feces of healthy women, an increased abundance of probiotic strains was not observed in the oral or vaginal samples, suggesting that *L. rhamnosus* GR-1 and *L. reuteri* RC-14 do not translocate from the gut to the vagina [26,52]. In contrast, another study showed that *Lactobacillus* spp. recover the asymptomatic or intermediate BV through the gut [53]. A different study reported that *Lactobacillus* spp. not only significantly altered the vaginal flora but also reduced the colonization of pathogenic microbes [54]. In the present study, a significant increase in *Lactobacillus* spp. was found in the CVF sample of the HNS group after LBP intake. By analyzing the sample dataset from the first, second, and third visits in the normal, intermediate, and BV groups, significant vaginal microbiota dynamics and LBP efficacy were found based on a comprehensive analysis of the normalization of BV levels in the HNS, which also showed a positive correlation between the alpha diversity and NS (Figure 3).

*Lactobacillus* spp. are known as potential biomarkers of a healthy vaginal microbiota, while vaginal dysbiosis can increase the colonization of anaerobic bacterial species associated with STIs, such as *Gardnerella, Prevotella, Sneathia, Ralstonia, Dialister,* and *Streptococcus* [17,35,55]. Herein, *Lactobacillus*, which was the dominant genera in the LNS group, showed a significant negative correlation with the NS, and genera related to BV, such as *Gardnerella, Streptococcus, Prevotella, Ralstonia*, and *Dialister*, showed a positive correlation [17]. In the present study of healthy women, it was found that the dominant genus in the vaginal microbiota was *Lactobacillus*, showing a negative correlation with alpha diversity (Figure 4). It also showed a negative correlation with the NS, which was related to *Gardnerella*. Alpha diversity showed the diversity of the vaginal microbiota, while NS represented the vaginal environment with some representative species. According to Wessels et al., high microbial diversity was observed in female sex workers regardless of the NS, suggesting that the NS may not represent vaginal microbial diversity [56]. Dols et al. suggested that microbial diversity in the vagina, like the NS, can be used to diagnose BV [57]. Therefore, it is believed that showing the vaginal microbiota for both markers will give a clearer indication of the healthy female flora. Although *Ureaplasma* is one of the genera most frequently associated with BV, which is further associated with complications, such as preterm birth, herein, a positive correlation of *Ureaplasma* only with Shannon diversity was observed [58]. Additionally, in our recent study, it was found that during pregnancy *Ureaplasma* and *Prevotella* colonization with *Lactobacillus* abundance facilitates term birth [59]. Thus, it might be possible that a high abundance of *Ureaplasma* with *Lactobacillus* in asymptomatic women carries a risk of preterm birth. Moreover, *Gardnerella* is normally present in the healthy vagina; consistently, a significant negative correlation was observed between *Gardnerella* with the NS [60]. However, a significant positive correlation of *Haemophilus*, which is significantly associated with obesity, with NS was observed [61]. Gestational obesity is associated with a greater risk of invasive *Haemophilus* infection and a poor pregnancy outcome [62]. STI-related bacteria, such as *Gardnerella, Atopobium, Prevotella, Streptococcus*, and *Ureaplasma*, can co-exist with *Lactobacillus* spp. and not cause any problems but can cause infections, such as BV, when the balance is disturbed [32]. In contrast, when *Lactobacillus* spp. and *Gardnerella* spp. co-existed, a negative correlation was observed between these two spp. that maintain vaginal health or susceptibility to pathogenic infections, respectively [63].

A shift in beta-diversity was confirmed following probiotic intake (Figure 5). Specifically, in Group B, the majority of samples showed a downward shift on the y-axis and a rightward shift on the x-axis. The vaginal environment often changes due to various factors, such as menstruation. As female hormone levels change, so does the vaginal environment. Although 12% (n = 3) of Group A’s LNS changed to an HNS at visit three, there was no significant change observed in alpha diversity (Figure 2A–C). Moreover, the taxa composition (Figure 2D,E) was also found to be different from that of Group B’s HNS. Considering that 88% (n = 26) of Group A did not experience a change in the LNS, and 60% (n = 6) of Group B changed to an LNS, it was considered that the probiotics may have a positive impact on maintaining and modifying the vaginal environment.

*Lactobacillus* species are considered as candidate probiotics for human health, including vaginal health [64,65]. Recent studies have shown the potential effects of a single specific species and/or combination of *Lactobacillus* species on dysbiotic conditions. In a previous study, oral intake of a combination of *L. rhamnosus* GR-1 with *L. reuteri* RC-14 during pregnancy was associated with a low risk for premature birth, which was directly associated with vaginal health and vaginal microbiota eubiosis [44]. In another study, the administration of probiotics, including the combination of 10 species, reduced the vaginal pH in women with an intermediate NS [66]. Here, the combination of *L. acidophilus* CBT LA1, *L. rhamnosus* CBT LR5, and *L. reuteri* CBT LU4 as an LBP was explored for vaginal dysbiosis. *L. acidophilus*, *L. rhamnosus*, and *L. reuteri* are used as raw materials in healthy functional foods (daily intake of 10^8^–10^10^ CFU) according to Health Functional Food Standards, Ministry of Food and Drug Safety. In another study, administering a 1:1 combination of *L. rhamnosus* IMC 501^®^ and *Lactobacillus paracasei* IMC 502^®^ (5 × 10^9^ CFU/capsule/day for 15 consecutive days) improved leucorrhea, itching, and vulvo-vaginal erythema/edema [29]. In the present study, *L. acidophilus, L. rhamnosus* and *L. reuteri* abundance showed a decrease at visit 2 (Figure 6). This may mean competition in the vaginal microbial community of *Lactobacillus* species. Figure 3 confirms that there was a change in the flora. At visits 2 and 3, there was a significant increase in *Lactobacillus* species abundance, such as for *L. acidophilus*, *L. rhamnosus*, and *L. reuteri*, suggesting that the vaginal environment was altered by lowering the pH through the secretion of lactic acid. This suggests an environment conducive to the settlement of *Lactobacillus* species. In addition, there was a tendency for *Lactobacillus* species abundance to increase between visit 1 and 3 through 6 weeks of intake, and a significant difference would be observed if intake occurred over a longer period of time.

Studies using probiotics have confirmed changes in vaginal dysbiosis as well as changes in nitrogen quality, vaginal secretion, odor, and NS [66]. Thus, consistent with the observations of the present study, consumption of LBPs is believed to inhibit an increase in alpha diversity and prevent vaginal dysbiosis in asymptomatic women. In addition, the results of short- and long-term interventions are dependent on the combination of spp. in the LBP. For example, short-term (1 week) intervention of *L. brevis* (CD2), *L. salivarius* subsp. *salicinius* (FV2), and *L. plantarum* (FV9) as an LBP resolved BV infections and improved vaginal health [67]. On the other hand, long-term (6 months) oral intake of *L. rhamnosus* GR-1 and *L. reuteri* RC-14 as an LBP did not enhance the curing of BV in Chinese women [68]. In the present study, improvement in the vaginal microbiota in women with LBP intake for just 6 weeks was observed. These findings suggested that the results of LBP intake may vary according to ethnicity, age, and lifestyle [12,69].

However, there were some limitations in this study. First, compared to 60% of participants who showed improvements, 40% with an HNS ≥ 4–10 as the abnormal group did not demonstrate improvement of vaginal dysbiosis. Therefore, STI-PCR, a multiplex PCR used to identify the presence of STI-related bacteria, including spp. of *Gardnerella*, *Ureaplasma*, and *Chlamydia*, was performed with third visit samples to assess the severity of the infection. An attempt was made to determine whether the levels of these species were reduced, but no significant results could be obtained. Consequently, additional research on the variations in the vaginal microbiota at a large scale is required. Second, even though the effect of LBP intake for a period of 6 weeks was validated, it was not possible to assess how long the effect would last once oral administration was stopped. Future studies should establish a prolonged study period and include longitudinal research. Finally, there was no placebo group in the final study to allow for comparisons. Moreover, subjects with vaginitis were excluded from the study. However, this study did not aim to investigate patients suffering from severe vaginitis, but rather those that were able to function normally. Therefore, the results of this study on the consumption of probiotics and their effects on the vaginal microbiota may be more relevant to healthy women.

The robust data produced in this study suggest that LBP consumption may assist with maintaining and increasing the population of *Lactobacillus* spp., as well as with maintaining and decreasing microbial diversity for asymptomatic vaginal dysbiosis in the short term. During the 6 week intake period, colonization of *Lactobacillus* spp. and a decrease in the NS were confirmed. This implied that LBP consumption enhanced the colonization of *Lactobacillus* spp. in the vagina via translocation from the gastrointestinal tract, as confirmed by qRT-PCR. Collectively, this microbiota-based study allowed the LNS and HNS vaginal microbiota to be distinguished in asymptomatic women, thereby facilitating the development of therapies and treatment options for vaginal dysbiosis. This study found that oral administration of an LBP containing *L. acidophilus* CBT LA1, *L. rhamnosus* CBT LR5, and *L. reuteri* CBT LU4 could promote vaginal health in asymptomatic women.

## Figures and Tables

**Table 1:** Clinical characteristics of asymptomatic women after Nugent scoring.

Parameters	Women with an LNS (n = 26)	Women with an HNS (n = 10)	*p* Value
Age (Median)	40.50 (11)	46.50 (11)	NSD
BMI (Median)	22.71 (3.71)	24.88 (4.86)	NSD
Gram stain (number of positive subjects)			
First visit	0	10	<0.05
Second visit	4	4	NSD
Third visit	3	4	NSD
Nugent score (Mean ± SD)			
First visit	0.81 ± 0.94	5.70 ± 1.83	<0.001 *
Second visit	1.54 ± 1.98	2.80 ± 2.44	<0.04 *
Third visit	1.69 ± 1.91	3.80 ± 3.33	NSD *

BMI, body mass index; LNS, Low Nugent score (LNS ≤ 3); HNS, High Nugent score (HNS ≥ 4–10); NSD, no significant difference. * Kruskal–Wallis test.

## Data Availability

The sequence data that support the findings of this study are available via BioProject, accession number PRJNA899123 (https://www.ncbi.nlm.nih.gov/bioproject/PRJNA899123, accessed on 12 April 2023).

## Supplementary Materials

The following supporting information can be downloaded at: https://www.mdpi.com/article/10.3390/nu15081862/s1, Figure S1: Changes in the microbial diversity at each visit based on the levels of different species in the vaginal microbiota. The principal component analysis shows the change in the beta diversity distance of the samples according to levels. Abbreviations: PCoA, principal component analysis; Table S1: qRT-PCR primers used in this study; Table S2: Composition of the reaction mixture for qRT-PCR and PCR thermal cycling conditions. References [70,71,72] are cited in the supplementary materials.
