# Supplementary material for: Lactobacillus Probiotics Improve Vaginal Dysbiosis in Asymptomatic Women
Source: Nutrients. 2023 Apr 13;15(8):1862. doi: 10.3390/nu15081862 (PMC10143682; doi:10.3390/nu15081862)
Supplement: Supplementary file 1 [file nutrients-15-01862-s001.zip › nutrients-2309525-supplementary.pdf]

Supplementary Materials:

The supplementary data to this article can be found online at:

Supplementary Table 1. qRT-PCR primers used in this study.

Target	Forward/ Reverse	Reference	Reference
L.acidophilus	Forward	GAAAGAGCCCAAACCAAGTGATT	70
	Reverse	CTTCCCAGATAATTCAACTATCGCTTA	
L.rhamnosus	Forward	CTAGCGGGTGCGACTTTGTT	71
	Reverse	GCGATGCGAATTTCTATTAT	
L. reuteri	Forward	CAGACAATCTTTGATTGTTTAG	72
	Reverse	GCTTGTTGGTTTGGGCTCTTC	

Supplementary Table 2. Composition of the reaction mixture for qRT-PCR and PCR thermal cycling conditions.

Final volume 20 μL		Amplification thermal condition		
DW	7 μL	95 °C	3 min	
Primer(F)	1 μL (10pmole)	95 °C	15 sec	45cycles
Primer(R)	1 μL (10pmole)	60 °C	15 sec	
Q-Mastermix (2X)	10 μL	72 °C	15 sec	
DNA	1 μL	4 °C	∞	

Abbreviations: DW, distilled water; F, forward; R, reverse.

Supplementary figure 1

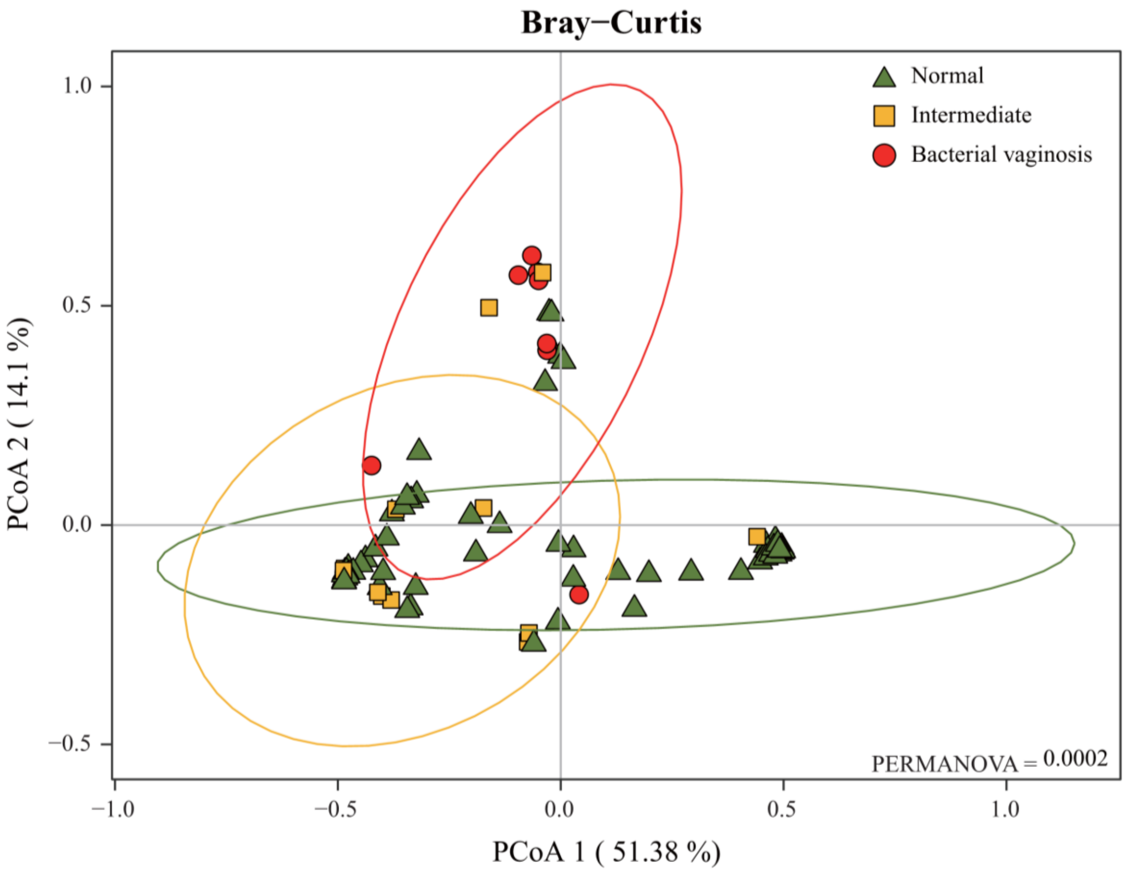

Supplementary Figure 1. Changes in microbial diversity according to the visit, based on the severity levels of the vaginal microbiota. Principal component analysis shows the change in the beta-diversity distance of the samples according to the severity levels. Abbreviations: PCoA, principal component analysis.
